# Life Expectancy in Marine Mammals Is Unrelated to Telomere Length but Is Associated With Body Size

**DOI:** 10.3389/fgene.2021.737860

**Published:** 2021-09-24

**Authors:** Kittisak Buddhachat, Janine L. Brown, Manthanee Kaewkool, Anocha Poommouang, Patcharaporn Kaewmong, Kongkiat Kittiwattanawong, Korakot Nganvongpanit

**Affiliations:** ^1^Department of Biology, Faculty of Science, Naresuan University, Phitsanulok, Thailand; ^2^Excellence Center in Veterinary Bioscience, Chiang Mai University, Chiang Mai, Thailand; ^3^Smithsonian Conservation Biology Institute, Center for Species Survival, Front Royal, VA, United States; ^4^Department of Veterinary Biosciences and Public Health, Faculty of Veterinary Medicine, Chiang Mai University, Chiang Mai, Thailand; ^5^Phuket Marine Biological Center, Phuket, Thailand

**Keywords:** age, body size, cetaceans, lifespan, sirenians

## Abstract

Marine mammals vary greatly in size and lifespan across species. This study determined whether measures of adult body weight, length and relative telomere length were related to lifespan. Skin tissue samples (*n* = 338) were obtained from 23 marine mammal species, including four Mysticeti, 19 Odontoceti and one dugong species, and the DNA extracted to measure relative telomere length using real-time PCR. Life span, adult body weight, and adult body length of each species were retrieved from existing databases. The phylogenetic signal analysis revealed that body length might be a significant factor for shaping evolutionary processes of cetacean species through time, especially for genus *Balaenoptera* that have an enormous size. Further, our study found correlations between lifespan and adult body weight (*R*^2^ = 0.6465, *p* < 0.001) and adult body length (*R*^2^ = 0.6142, *p* ≤0.001), but no correlations with relative telomere length (*R*^2^ = −0.0476, *p* = 0.9826). While data support our hypothesis that larger marine mammals live longer, relative telomere length is not a good predictor of species longevity.

## Introduction

Marine mammals consist of cetaceans (mysticetes and odontocetes), sirenians (manatees and dugong), pinnipeds (phocids, otarids, and walrus), marine and sea otters, and the polar bear (Jefferson et al., 1994). These species are not randomly distributed across the world’s oceans, but rather occupy species-specific geographical niches that vary by depth, temperature, and food resources (Jefferson et al., 1994). Many of these species are at risk of extinction (Reynolds et al., 2009), and it has been estimated that nearly three-quarters of all marine mammals experience high levels of human interference, with the most at-risk species inhabiting coastal areas in close proximity to humans (Davidson et al., 2012). Today, a large number of species living in more open waters, which generally have been considered safe from anthropogenic impact, also are being negatively affected by human activities (McCauley et al., 2015). The greatest direct threats to marine mammals are incidental catch (particularly by fisheries), pollution, commercial harvest, hunting, and vessel strikes (Avila et al., 2018). Indirect threats include degradation of habitats and food sources that increase disease susceptibility and reduce reproductive rates, all of which negatively impact population survival (Schipper et al., 2008; Simmonds et al., 2014). Conservation efforts *in situ* have had mixed results despite their visibility and high value. For example, according to a 2019 report (Valdivia et al., 2019), of 62 marine mammal and sea turtle species, 18 mammal (78%) and six turtle (75%) populations increased after being listing by the United States Endangered Species Act (ESA), while two marine mammal (9%) populations declined and three mammal (13%) and two turtle (25%) populations showed no change after ESA protection. And while some marine mammal species breed well in captivity [e.g., bottlenose dolphin (*Tursiops truncatus*) (Jaakkola and Willis, 2019), Indo-Pacific bottlenose dolphins (*Tursiops aduncus*) (Koga et al., 2019)], which can be a hedge against extinction in the wild, most do not. Thus, life history data from captive bred and wild animals are important for risk modeling, although it is limited for most marine mammal species (Davidson et al., 2012; IUCN, 2020).

In general, marine mammals are fairly long-lived (Bourg and Bourg, 2020), but determining age is difficult, particularly for those that live fully underwater. Births and deaths are rarely observed in the wild, so reliable population demographics are lacking for most species. A number of methods for age estimation have been tested in marine mammals, including measures of tissue amino acid racemization (Garde et al., 2007, 2012), fatty acid signatures from blubber, bone mineral density (Bourg and Bourg, 2020), counting dentine growth layer groups (GLGs) in bone or teeth (Read et al., 2018; Bourg and Bourg, 2020), radiographic imaging to determine dentine thickness (Barratclough et al., 2019), measures of telomere length (Olsen et al., 2018; Whittemore et al., 2019) and epigenetic (Polanowski et al., 2014). Estimated lifespans of 211 years in bowhead whale (*Balaena mysticetus*) determined by aspartic acid racemization in the eye lens (George et al., 1999), 73 years in dugong (*Dugong dugon*) measured by GLG counts in incisors (Marsh, 1980), and 110 years in the southern hemisphere blue whale (*Balaenoptera musculus*) and 114 years in the fin whale (*Balaenoptera physalus*) based on counts of dark and light areas in the core of ear plugs (Ohsumi, 1979) have been reported.

More recently, relative telomere length (rTL) measurements by quantitative real-time polymerase chain reaction (qPCR) have been gaining attention for age estimation (Cawthon, 2002; Hewakapuge et al., 2008; O’Callaghan et al., 2008; Heidinger et al., 2012; Buddhachat et al., 2017; Eastwood et al., 2019; Kaewkool M.K.W. et al., 2020). This method was first used by Cawthon (2002) and based on amplification DNA portion of telomeric repeats TTAGGG that share a conserved sequence of six base 5′-TTAGGG-3′ at the terminal end of vertebrate chromosome. The validity of rTL measures by qPCR has been demonstrated by comparisons with standard methods, such as terminal restriction fragment length (TRF) in humans of known ages (5–94 years), exhibiting a high correlation (*R*^2^ = 0.68) (O’Callaghan et al., 2008). In zebra fish, rTL decreases with increasing age through telomere attrition (Heidinger et al., 2012). Among bird species of varying longevity, initial rTL was related to lifespan, as well as to lifetime reproductive success (Eastwood et al., 2019). However, Whittemore et al. (2019) has proposed it is not initial telomere length that correlates with longevity across a wide range of mammals, but rather the rate of telomere attrition.

This study investigated relationships and phylogenetic signals between rTL in skin samples of 23 species of marine mammals found along Thailand sea coasts (Andaman Sea and Gulf of Thailand) and measures of body weight and length and degree to which they may be used to estimate maximum life span.

## Materials and Methods

### Samples and Metadata of Traits From Some Cetaceans

This study analyzed 338 skin samples from 23 Sirenia and Cetacea species (Table 1) from the Phuket Marine Biological Center, Phuket, Thailand. According to the Animals for Scientific Purposes Act, B.E. 2558 (2015), because a part of this experiment was performed on carcasses of stranded marine mammals, no ethical approval was required for this study, which was confirmed by the Animal Ethics Committee, Faculty of Veterinary Medicine, Chiang Mai University (License number U1006312558). Information on maximum lifespan, adult weight and length was obtained from several publicly available databases as shown in Table 1 (Francis and Barrett, 2008; Stuart and Stuart, 2015; Cerchio and Yamada, 2018; Tacutu et al., 2018; Myers et al., 2020; National Oceanic and Atmospheric Administration within the Department of Commerce, 2020).

**TABLE 1:** Maximum lifespan, adult body weight and length of Sirenia and Cetacea samples used in this study.

Number	Common name	*Scientific name*	Accession number	N	Maximum lifespan (Year)	Adult body weight (Kg)	Adult body length^*e*^ (m)
	**Sirenia**						
1	Dugong	*Dugong dugon*	KJ022758	128	73^*a*^	360,000.0^*a*^	3.00^*e*^
	**Cetacea**						
	Mysticeti						
	**Family** Balaenopteridae						
2	Blue whale	*Balaenoptera musculus*	JN801062	1	110^*a*^	136,000.0^*a*^	23.00^*e*^
3	Bryde’s whale	*Balaenoptera edeni*	KY938508	5	72^*a*^	16,000.0^*a*^	13.00^*e*^
4	Omura’s whale	*Balaenoptera omurai*	AB116097	4	95^*b*^	2,500.0^*a*^	11.00^*f*^
	Odontoceti						
	**Family** Delphinidae						
5	Common bottlenose dolphin	*Tursiops truncatus*	MF669486	1	51.6^*a*^	200.0^*a*^	2.95^*e*^
6	Fraser’s dolphin	*Lagenodelphis hosei*	AB610384	6	18^*c*^	164.0^*a*^	2.50^*e*^
7	Indo-Pacific bottlenose dolphin	*Tursiops aduncus*	KY542107	26	34^*b*^	280.0^*a*^	2.60^*e*^
8	Indo-Pacific humpback dolphin	*Sousa chinensis*	KX364256	4	40^*d*^	265.0^*d*^	5.20^*e*^
9	Irrwaddy dolphin	*Orcaella brevirostris*	MG703251	2	30^*a*^	190.0^*a*^	2.15^*e*^
10	Common dolphin	*Delphinus delphis*	U02664	2	40^*a*^	100.0^*a*^	2.50^*e*^
11	Pantropical spotted dolphin	*Stenella attenuata*	KY542112	29	46^*a*^	112.5^*a*^	2.50^*e*^
12	Rough-toothed dolphin	*Steno bredanensis*	AY842471	7	32^*a*^	114.0^*a*^	2.30^*e*^
13	Spinner dolphin	*Stenella longirostris*	NC032301	34	26^*d*^	51.5^*d*^	1.80^*e*^
14	Striped dolphin	*Stenella coeruleoalba*	AM498734	57	57.5^*a*^	112.5^*a*^	2.25^*e*^
15	False killer whale	*Pseudorca crassidens*	AB377526	8	62.5^*a*^	748.0^*a*^	3.60^*e*^
16	Risso’s dolphin	*Grampus griseus*	AM498741	4	42.5^*a*^	425.0^*a*^	3.80^*e*^
17	Short-finned pilot whale	*Globicephala macrorhynchus*	AJ226120	1	63^*a*^	2,200.0^*a*^	4.15^*e*^
	**Family** Kogiidae						
18	Dwarf sperm whale	*Kogia sima*	NC041303	4	22^*a*^	202.5^*d*^	2.40^*e*^
19	Pygmy sperm whale	*Kogia breviceps*	KY542109	3	17^*a*^	424.6^*a*^	3.05^*e*^
	**Family** Phocoenidae						
20	Finless porpoise	*Neophocaena phocaenoides*	MG719601	5	33^*a*^	32.5^*a*^	1.75^*e*^
	**Family** Physeteridae						
21	Sperm whale	*Physeter macrocephalus*	M93154	5	77^*a*^	28,500.0^*a*^	11.00^*e*^
	**Family** Ziphiidae						
22	Blainville’s beaked whale	*Mesoplodon densirostris*	KF032863.2	1	27^*d*^	925.0^*d*^	4.70^*e*^
23	Cuvier’s beaked whale	*Ziphius cavirostris*	AB610404	1	62^*a*^	2,701.0^*a*^	6.50^*e*^

*^*a*^AnAge database (Tacutu et al., 2018).*

*^*b*^Stuarts’ Field Guide to the Larger Mammals of Africa (Stuart and Stuart, 2015).*

*^*b*^NOAA Fisheries Web: https://www.fisheries.noaa.gov/ (National Oceanic and Atmospheric Administration within the Department of Commerce, 2020).*

*^*c*^Animal Diversity Web: https://animaldiversity.org/ (Myers et al., 2020).*

*^*d*^Guide to the Mammals of Southeast Asia (Francis and Barrett, 2008).*

*^*e*^Encylopedia of Marine Mammals (Cerchio and Yamada, 2018).*

### DNA Extraction and Real-Time PCR

Skin samples (2 × 2 cm) were preserved in 95% ethanol for DNA extraction according to manufacturer’s instructions (DNeasy Blood and Tissue Kit, QIAGEN, Germany). DNA, diluted to 50 ng/μl, was measured qualitatively and quantitatively using agarose gel electrophoresis, and absorbance at A260, respectively. To estimate the telomere length of individual samples, qPCR was carried out using Eco Real-Time PCR System (Illumina, United States) (Hewakapuge et al., 2008; Buddhachat et al., 2017; Kaewkool M. et al., 2020). Briefly, DNA amplification by qPCR consisted of 1X real time master mix (Bioline, England), 270 nM of telomere primer of tel 1, 5′-GGTTTTTGAGGGTGAGGGTGAGGGTGAGGGTGAGGGT-3′, 900 nM of tel 2, 5′-TCCCGACTATCCCTATCCCTATCCC TATCTATCCCTA-3′ and 50 ng of extracted DNA in a total volume of 10 μl. Additionally, the single copy gene 36B4 was used as a control and amplified using 400 nM of the forward primer 5′-CAGAGTGAYGTGCAGCTGAT-3′, and for reverse primer 5′-AAGCACTTCAGGGTTGTAGATGCTGCC-3′ to normalize the copy of telomere for inter-individual comparison. The cycling profile for the telomere (T) PCR was as follows: 40 cycles of 95°C for 15 s, and annealing temperature for each species (Table 2) for 2 min. For the 36B4 single copy gene (S), the reaction was conducted with 30 cycles of 95°C for 15 s, and annealing temperature per species for 1 min (Table 2). The cycle threshold (*C*_*t*_) values acquired from qPCR were used for analyzing the rTL through the following formula: 2^[C*t(telomere)*–*Ct(*36*B*4 gene)]^ and was expressed as T/S or rTL (Livak and Schmittgen, 2001). The measurement of rTL by qPCR has been validated for reliability, and shown that higher rTLs are found in long-lived animals (Cawthon, 2002; Buddhachat et al., 2017; Eastwood et al., 2019).

**TABLE 2:** Annealing temperature of telomere primer and single copy gene primer.

	Annealing temperature (°C)	
	
Common name	*Telomere* gene	*36B4* gene
Dugong	65.0	65.5
Blue whale	64.6	68.0
Bryde’s whale	64.0	68.0
Omura’s whale	61.9	67.8
Common bottlenose dolphin	61.9	65.5
Fraser’s dolphin	65.0	65.5
Indo-Pacific bottlenose dolphin	61.9	65.5
Indo-Pacific humpback dolphin	65.0	68.0
Irrwaddy dolphin	64.6	68.0
Common dolphin	63.1	68.0
Pantropical spotted dolphin	64.0	67.0
Rough-toothed dolphin	65.0	67.0
Spinner dolphin	65.0	68.0
Striped dolphin	65.0	65.5
False killer whale	60.4	68.0
Risso’s dolphin	63.1	67.0
Short-finned pilot whale	64.6	65.5
Dwarf sperm whale	64.6	68.0
Pygmy sperm whale	65.0	68.0
Finless porpoise	64.6	65.5
Sperm whale	64.6	67.8
Blainville’s beaked whale	63.1	65.7
Cuvier’s beaked whale	64.6	68.0

### Phylogenetic Signal Analysis

A phylogenetic tree of a sirenian and 22 cetaceans was constructed based on the sequences of control regions (trimmed for approximately 220 bp) retrieved from GenBank (Table 1) and aligned using MEGA X (Kumar et al., 2018). The phylogeny was built through Bayesian inference under MrBayes 3.2 (Ronquist and Huelsenbeck, 2003) with the appropriate substitution model and corrected by Akaike Information Criterion (AIC) obtained from jModelTest2 (Nylander, 2004) as GTR + Γ (a General Time Reversible and a gamma-shaped distribution of rates across site). The Bayesian inference was run with two independent searches with random starting trees for 1,000,000 generations, in which the diagnostic was calculated every 1,000 generations and compared using four Markov chain Monte Carlo chains (temp = 0.2). The log-likelihood scores were used for plotting the convergence in Tracer v1.5 (Rambaut et al., 2013) and building a strict consensus tree with dugong as the outgroup, which was completed by removal of the first 25% of the generations from each run. After obtaining the phylogeny of cetaceans, a phylogenetic signal was tested to determine if the traits (i.e., lifespan, body weight, body length, and relative telomere) were similar within closely related species using Blomberg’s *K* and Pagel’s λ under function phyloSignal in package phylosignal (Keck et al., 2016). K and λ show the level of relatedness between phylogeny and traits in evolutionary process under Brownian motion (BM), namely phylogenetic signal. The *K* and λ = 0 represents no phylogenetic signal, *K* and λ = 1 suggests that a trait was evolved according to BM model of evolution (i.e., gradual, random, non-directional trait change through time) (Felsenstein, 1985), and *K* and λ > 1 indicates stronger resemblance among closely related species than expected under BM. We used Local Indicators of Phylogenetic Association (LIPA) by Local Moran’s I (li) through function lipaMoran in package phylosignal to describe local traits patterns or detect hotspots of autocorrelation for determining whether traits evolve similarly into the phylogeny (Keck et al., 2016).

### Statistical Analysis

Life history information of the marine mammal species in this study that included lifespan, log(body weight), body length, and log(rTL) are shown by heatmaps, circle graphs, bar plots, and box plots, respectively. Relationships with lifespan were determined by general linear model (GLM) univariate analyses including the variables rTL, log(rTL), body weight, log(body weight), body length, or log(body length), and for rTL with variables including body weight, log(body weight), body length, or log(body length). Relationships between lifespan and other variables including rTL, body weight, log(body weight), body length, log(body length) were further evaluated by general multivariate linear models. The most suitable model for estimating life span was chosen by the Akaike information criterion (AIC) value through *R* program. The selected models with the lowest AIC were tested for prediction accuracy by coefficient of determination (*R*^2^) (Alexander et al., 2015).

## Results

### Telomere Assay

In the present study, rTL was estimated by real-time PCR, expressed as the ratio of telomere to reference gene (single copy gene) for each individual and measured in triplicate. The precision of the assay based on average coefficient of variation (CV) values of each individual was 40%, demonstrating relatively high intra-individual variation and low repeatability of the assay (data not shown).

### Phylogenetic Signals and Trait Divergences Across Cetaceans

**FIGURE 1:**
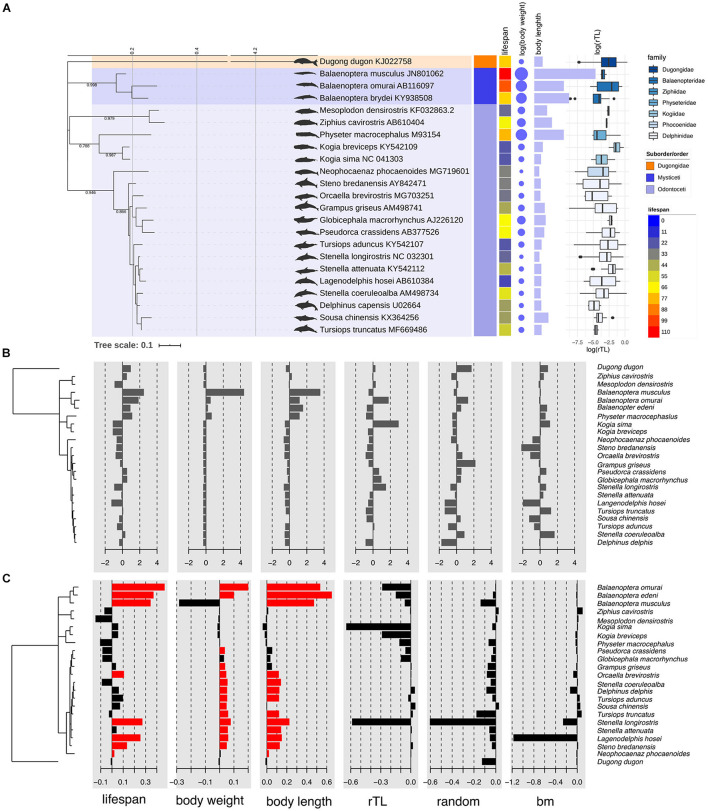
Phylogenetic tree of marine mammals based on sequences of control regions with information about average lifespan, body weight, body length, and relative telomere length of each species found in Thailand oceans **(A)**. Phylogenetic tree with value of each trait centered and scaled **(B)** and with Local Moran’s index (*I*_*i*_) values for each species for traits including lifespan, body weight, body length, relative telomere length (rTL), random dataset, and Brownian Motion (BM) dataset **(C)**. The red bar indicates the significant *I*_*i*_ values at *p* < 0.05. The phylogeny was built based on 220 pb control region of 22 cetacean species under GTR + Γ through Bayesian inference. Dugong was rooted.

To determine if traits (lifespan, body weight, body length obtained from metadata and rTL) of 22 cetacean species were similar within closely related species or the same clade, a phylogentic signal based on Blomberg’s *K* and Pagel’s λ was calculated as shown in Table 3. We found that lifespan and body length showed a significant phylogenetic signal for Blomberg’s *K* (*K* > 0, *p* < 0.05), whereas for Pagel’s λ, lifespan, body weight and body length indicated a high phylogenetic signal (λ > 0, *p* < 0.05). By contrast, rTL did not show phylogeny across cetacean species. As illustrated in Figures 1B,C, three closely related species belonging to genus *Balaenoptera* tended to share similar trait values, especially lifespan, body length and body weight. By contrast, two adjacent clades differed, except for *Physeter macrocephaslus*, which has a large body size and long life expectancy similar to *Balaenoptera*. The hotspot of autocorrelation across traits was assessed by local Moran’s *I* for each species into the phylogeny of cetacean species (Figure 1). LIPA analysis exhibited remarkable local positive autocorrelation in two clades: the genus *Balaenoptera* with high values for lifespan, body weight and body length, and the majority of family *Delphinidae* (genus *Orcaella*, *Tursiops*, *Stenella*, *Sousa*, *Delphinus*, and *Lagenodelphis*, *Steno*) with low values for lifespan, body weight and body length (Figure 1). However, there were no species with hotspot autocorrelations for rTL.

**TABLE 3:** Phylogenetic signal for traits based on the phylogenetic tree of 22 cetacean species.

Traits	Blomberg’s *K*	*p*-value	Pagel’s λ	*p*-value
lifespan	**0.53**	0.0030	**0.96**	0.0259
body weight	0.66	0.0810	**1.04**	0.0010
body length	**1.10**	0.0030	**1.05**	0.0010
relative telomere length (rTL)	0.16	0.8700	0.00	1.0000
random	0.11	0.9560	0.28	1.0000
Brownian motion	0.12	0.9670	0.00	1.0000

*Trait value with significant difference at *p* < 0.05 are in bold.*

### The Relationships of Each Traits for Cetacean Species

**FIGURE 2:**
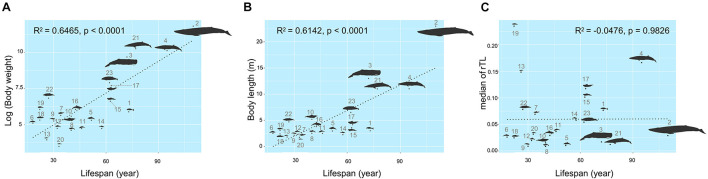
Relationships of maximum lifespan with body weight **(A)**, body length **(B)**, and median relative telomere length **(C)** across marine mammal species found in Thailand oceans. The size of the figures illustrates accordance to the real ratio. The numbers represent species of marine mammals as depicted in Table 1.

**FIGURE 3:**
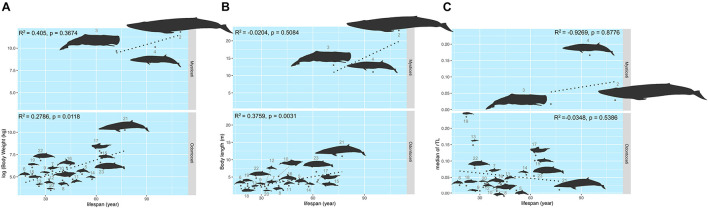
The relationship of the maximum lifespan with log (body weight) **(A)**, body length **(B)**, and median rTL **(C)** across Mysticeti and Odonotoceti. The size of the figures illustrates in accordance to the real ratio. The numbers represent species of marine mammals as depicted in Table 1.

The maximum lifespan of the marine mammals in this study ranged from 18 to 110 years, with *L. hosei* having the shortest and *B. musculus* the longest longevity (Figure 1A). Based on morphometrics (body weight and length), *B. musculus* was the largest, while *L. hosei* was the smallest species (Figures 1, 2). In addition, baleen whales were larger in size (both in body weight and length) than species belonging to *Odontoceti* in parallel with longer maximum lifespans. Body sizes, both weight and length, were positively correlated with maximum lifespan, with adjusted *R*^2^ = 0.6465 (*p* < 0.0001) and 0.6142 (*p* < 0.0001), respectively (Figures 2A,B). Median rTL values revealed variation across species (Supplementary Table 1), with the highest and lowest values observed in *K. breviceps* (rTL = 0.24, maximum lifespan = 22) and *D. delphis* (rTL = 0.008, maximum lifespan = 40), respectively, but no relationship between maximum lifespan and median rTL noted (adjusted *R*^2^ of −0.0476, *p* = 0.9826) (Figures 1B,C, 2B). As depicted in Figure 3, relationships between maximum lifespan and median rTL of species within *Mysticeti* or *Odontoceti* also were not observed (Figure 3C). However, maximum lifespan of species within the *Odontoceti* was associated with log body weight and length, with an adjusted *R*^2^ = 0.2786 (*p* = 0.0118) and 0.3759 (*p* = 0.0031), respectively (Figures 3A,B).

The mixed model used for creating a model for lifespan prediction of marine mammals is shown in Table 4. Log-transformed data of body weight was the best univariate model, with AIC = 192.26 and adjusted *R*^2^ = 0.6465 (*p* < 0.0001) (Table 4). The best multivariable model included the log of body weight and the untransformed body length (AIC = 193.04, adjusted *R*^2^ = 0.6479), given a function as lifespan = 5.839 × log(body weight) + 1.543 × body length + 4.102 (Table 3). Notably, median rTL had no statistical significance in any models.

**TABLE 4:** Mixed model for average lifespan using body weight, body length and median rTL for estimating average lifespan.

Variable	d.f.	AIC	Adjusted *R*^2^	*p*-value	Estimate of coefficients	The best model for life span estimation	
						
						Lifespan ∼ Log(body weight)	Lifespan ∼ Log(body weight + body length)
univariate analyse: lifespan ∼ a variable					log(body weight)	9.039 ± 1.408	5.839 ± 3.373
rTL	21	217.25	–0.0476	0.9826	body length	-	1.543 ± 1.479
log(rTL)	21	217.25	–0.0431	0.7662	intercept	−8.490 ± 9.510	4.102 ± 15.354
body weight	21	202.80	0.4410	0.0063			
log(body weight)	21	192.26	0.6465	<0.0001			
body length	21	194.25	0.6145	<0.0001			
log(body length)	21	193.83	0.6216	<0.0001			
univariate analyse: rTL ∼ a variable							
body weight	21	–60.09	–0.0390	0.6805			
log(body weight)	21	–60.03	–0.0415	0.7280			
body length	21	–60.04	–0.0414	0.7263			
log(body length)	21	–59.91	–0.0470	0.9119			
multivariate analyse: life span ∼ variables							
rTL, log(body weight)	20	194.03	0.6285	<0.0001			
rTL, body length	20	195.98	0.6000	<0.0001			
log(body weight), body length	20	193.04	0.6479	<0.0001			
rTL, log(body weight), body length	19	195.03	0.6296	<0.0001			

## Discussion

In the present study, our results revealed two significant findings: (i) lifespan and body size appeared to be phylogenetically conserved, especially body length, based on Blomberg’s *K* and Pagel’s λ = 1; and (ii) lifespan is likely to be connected to body size, both weight and length, but not to median rTL. This information can provide insight into the drivers of conserved traits in the evolutionary process of cetacean species, as well as relationships among life variables that might contribute to longevity.

The phylogeny of 22 cetaceans and traits including lifespan and body sizes (weight and length) gave statistically significant phylogenetic signals using both Blomberg’s *K* and Pagel’s λ. *K* and λ-value for body length showed phylogenetic conservatism (*K* = 1.10 and λ = 1.05) corresponding to evolution processes superior to the BM model (e.g., genetic stabilizing and genetic drift). In addition, LIPA analysis revealed that the genus *Balaenoptera* showed a considerable hotspot autocorrelation for body length. This was consistent with a study of Slater that explained the independent evolution of mysticete body size might be driven by ocean dynamics like wind-driven upwellings, with high prey density as the primary determinant of efficient foraging of baleen whales (Slater et al., 2017). The ecological prey scape might be an important driver rather than predatory avoidance and niche partitioning (Slater et al., 2017). A phylogenetic signal of λ-value for lifespan and body weight in cetacean species was also significant, but lower than that for body length. This suggested that those two traits might be more rapid diversification than body length causing the relatively variation among the closely related species (Smith et al., 2004; Kamilar and Cooper, 2013). The relative variation in lifespan and body weight were noted in the closely related species of family *Delphinidae*. However, several studies found similar body size among congeneric species or within the same clade [e.g., primates (Kamilar and Cooper, 2013), mammals (Smith et al., 2004)]. For the study of phylogenetic signals in primate traits, lifespan showed a low phylogenetic signal (Kamilar and Cooper, 2013) in contrast to marine mammal lifespans in the present study, which had a moderate, but significant phylogenetic signal. This might represent a difference in ecological niches between terrestrial and marine mammals affecting the extrinsic mortality risk. A study by Healy et al. (2014) demonstrated that, in addition to the positive correlation between lifespan and body size, there was a striking difference between flying and non-flying vertebrates related to maximum lifespan, as birds with similar body sizes to non-flying vertebrates live longer. Thus, the volant ability and enormous size appear to be important traits for investment in long-term evolution because they reduce the risk of extrinsic mortality (Healy et al., 2014; Slater et al., 2017; Whittemore et al., 2019).

It was not surprising, however, that the rTL in marine mammals was not a good phylogenetic signal due to the presence of high variation of rTL within the same clade, indicating non-directional change or random rTL values through time within mammal marines. For example, the lifespan of *K. breviceps* (*n* = 3) was 22 years and had median rTL of 0.23, whereas *B. musculus* (*n* = 1) with the longest lifespan (110 years) had a median rTL of 0.03. The rTL variation observed across different species in this study may be due to (i) the limited number of specimens for some species, (ii) the uneven distribution of age classes within species, and/or (iii) inaccurate estimates of true lifespans (Magalhães et al., 2007), (iv) the variation of telomere assays, or (v) the variation of rTL within intra-individual and low repeatability (Olsen et al., 2012). Quality control for rTL estimates using real-time PCR is important in identifying a suitable assay with high consistency, accuracy, and resolution (Olsen et al., 2012). While our findings showed that there were no significant relationships between median rTL and lifespan across marine mammal species, the lifespan of Odontoceti species actually appeared to be inversely related to median rTL. A study of telomeres in 60 different mammals demonstrated that species with long telomeres have shorter lifespans, which is in agreement with marine mammal data (Gomes et al., 2011). It was suggested that median rTL might be not be a major factor in determining lifespan in these groups. Further, a wide variety of bird and mammal species has been studied with no strong correlations found between lifespan and initial telomere length, although there were significant relationships between telomere shortening rate and lifespan of a species (Whittemore et al., 2019). Several other studies also have reported correlations between telomere attrition and a species’ lifespan, including in birds (Haussmann et al., 2003; Boonekamp et al., 2014; Tricola et al., 2018), chimpanzees (Tackney et al., 2014), and cynomolgus monkeys (Lee et al., 2002). However, one study in zebra finches clearly showed that initial telomere length measured at 25 days was reliable for lifespan estimation of individuals (Heidinger et al., 2012).

In the present study, we were not able to measure absolute telomere length, and so had to rely on estimate ages for some cetacean species with limited demographic data (Taylor et al., 2007); i.e., based on five life history parameters obtained from 58 cetacean species. A one-point-in-time measure for rTL was determined from carcasses, contributing to an inability to estimate either initial telomere length or rate of shortening as predictors of longevity.

Relationships between genetically related species were empirically created using a phylogenetic tree annotated with datasets of body index and median rTL. The phylogenetic tree was created based on partial control region sequences of 23 marine mammal species inhabiting Thailand oceans and consisted of a sirenian (*D. dugon*) and six families of cetaceans, including *Balaenopteridae* (three species), *Ziphiidae* (two species), *Physeteridae* (one species), *Kogiidae* (two species), *Phocoenidae* (one species), and *Delphinidae* (14 species). Thailand accounts for a quarter of cetacean species worldwide (Jefferson et al., 2015), and so represents a highly diverse region for marine mammals. *Balaenopteridae* members showed a larger body size than the others, but median rTL appeared not to relate. We suggest that each species may undergo specific evolutionary changes with respect to aging in response to their ecological habitat, behavior, and intrinsic factors (heart rates, metabolic rates), which then leads to different median rTL and maximum lifespan relationships.

Measuring telomere shortening in cetaceans is challenging owing to the difficulty of collecting samples from free-ranging animals, and the limited number of species held in captivity. Thus, other traits such as average body weight and body length were used to correlate with maximum lifespan and found that larger species like baleen whales were longer-lived, whereas species of *Odontoceti* that had a smaller size also had a shorter lifespan. Similarly, *P. macrocephalus* belonging to *Odontoceti* has a larger mass and long lifespan (77 years). Moderate correlations (>0.6) between lifespan and log-transformed body weight and/or body length were observed for all species in this study. Our findings further suggest that body size, including body weight and length, is a factor associated with species lifespan in accordance with the results of Magalhães et al. (2007). Ecological factors or the absence of predators likely drive larger animals to have longer lifespans (Kirkwood and Austad, 2000; Magalhães et al., 2007). In addition, higher heart rates are associated with shorter lifespans, in addition to increased risks of cardiovascular mortality through processes involving protein oxidation, free radical production, inflammation, and telomere shortening (Levine, 1997; Zhang and Zhang, 2009). Membrane composition might also be linked to longevity, with longer lifespans being associated with more saturated and monounsaturated fatty acids membrane structures (Hulbert, 2008). More recently, it has been shown that mitochondrial DNA (mtDNA) GC content exhibits significant correlations with maximum lifespan (MLS), and when included as a variable with body mass to predict the MLS is highly predictive (Lehmann et al., 2008, 2013; Toren et al., 2016). MtDNA GC content based on MitoAge database (Toren et al., 2016) might be used as a determinant factor to increase the predictive power to estimate lifespan of marine mammals. Animals such as the bowhead whale (*B. mysticetus*) and gray whale (*Eschrichtius rubustus*) are recognized as among the top 1% of longest-lived mammals, possibly due to adaptive mechanisms of cell physiology that support cell survival under extreme environmental conditions, such as DNA maintenance and repair, ubiquitination, apoptosis, immune responses, and insulin signaling (Seim et al., 2014; Keane et al., 2015; Toren et al., 2020). In addition, a study by Yanai et al. (2017) revealed that longevity-associated genes (LAGs), especially Sod2, Sirt1, Mtor, and Rps6 kb1, that have a high number of orthologs are overrepresented across diverse taxa with a high evolutionary distance (yeast, worm, fruit fly, and mouse) and may affect the extension of lifespan.

Finally, longevity may be related to survival tactics and how susceptible species, large and small, are to predation (Ballance, 2018). Dolphins and porpoises with a small body size are likely to be maimed or killed by larger-bodied sharks (Ballance, 2018) and so do not live as long, whereas baleen and larger toothed whales such as *P. macrocephalus* live longer than smaller dolphins and porpoises because they are less susceptible to predation. Species with a higher likelihood of survival may have evolved to age more slowly (Kirkwood and Austad, 2000).

## Conclusion

Our study revealed two significant findings that: (i) lifespan and body length appear to be important traits involved in the evolutionary process of cetacean species, particularly the genus *Balaenoptera*; and (ii) median rTL of 23 marine mammal species was not correlated with maximum lifespan of individual species, whereas body size (body weight and length) was. This study was limited by a lack of basic information on maximum lifespans of cetacean species retrieved from secondary sources (e.g., text book and online databases), and because they are free-ranging in habitats that are difficult to monitor. However, we attempted to use data from accredited sources as much as possible to compare across species, and provide the first comparative analysis of how life traits may be related to longevity. A one-point-in-time measure of rTL to obtain the median rTL was not a good predictor for longevity, which was not unexpected given the limitations of that measure and findings in other studies. We believe that the enormous size of *Balaenoptera* species likely reduces the extrinsic mortality risk from environmental pressures (e.g., predators). Finally, our model could be used for lifespan prediction of other marine mammal species based on basic morphometric data.

## Data Availability Statement

The datasets presented in this study can be found in online repositories. The names of the repository/repositories and accession number(s) can be found in the article/Supplementary Material.

## Ethics Statement

Ethical review and approval was not required for the animal study because according to the Animals for Scientific Purposes Act, B.E. 2558 (2015), since a part of this experiment was performed on carcass of stranding marine mammals, no ethical approval was required for this study and confirmed by the Animal Ethics Committee, Faculty of Veterinary Medicine, Chiang Mai University (License number U1006312558).

## Author Contributions

KB assisted in conducting the experiments, performed the statistical analysis, and data visualization and wrote the manuscript. JB assisted in discussion of the data and edited the manuscript. MK and AP performed the telomere experiments. PK and KK supported the tissue and preliminary data of marine mammals. KN designed and conducted all of the experiments and wrote the manuscript. All authors have read and approved the final manuscript.

## Conflict of Interest

The authors declare that the research was conducted in the absence of any commercial or financial relationships that could be construed as a potential conflict of interest.

## Publisher’s Note

All claims expressed in this article are solely those of the authors and do not necessarily represent those of their affiliated organizations, or those of the publisher, the editors and the reviewers. Any product that may be evaluated in this article, or claim that may be made by its manufacturer, is not guaranteed or endorsed by the publisher.
