# Supplementary material for: Life Expectancy in Marine Mammals Is Unrelated to Telomere Length but Is Associated With Body Size
Source: Front Genet. 2021 Sep 24;12:737860. doi: 10.3389/fgene.2021.737860 (PMC8498114; doi:10.3389/fgene.2021.737860)
Supplement: Supplementary file 1 [file Data_Sheet_1.DOCX]

**Supplemental table 1**

The average and variation in rTL of 22 cetaceans and dugong using realtime PCR.

| Common Name | Scientific Name | mean | SD |
| --- | --- | --- | --- |
| Blainville's beaked whale | *Mesoplodon densirostris* | 0.07351158 | 0.0156309 |
| Blue whale | *Balaenoptera musculus* | 0.03541297 | 0.0156309 |
| Bryde's whale | *Balaenoptera edeni* | 0.02655811 | 0.04836576 |
| Common bottlenose dolphin | *Tursiops truncatus* | 0.00850877 | 0.004696 |
| Cuvier's beaked whale | *Ziphius cavirostris* | 0.0575271 | 0.00725031 |
| Dwarf sperm whale | *Kogia sima* | 0.05334175 | 0.06608391 |
| False killer whale | *Pseudorca crassidens* | 0.15513657 | 0.13654903 |
| Finless porpoise | *Neophocaena phocaenoides* | 0.05249906 | 0.07769608 |
| Fraser's dolphin | *Lagenodelphis hosei* | 0.12542536 | 0.16429204 |
| Indo-Pacific bottlenose dolphin | *Tursiops aduncus* | 0.20616252 | 0.27455121 |
| Indo-Pacific humpback dolphin | *Sousa chinensis* | 0.04685127 | 0.05077129 |
| Irrwaddy dolphin | *Orcaella brevirostris* | 0.06870793 | 0.04350501 |
| Long-beaked common dolphin | *Delphinus delphis* | 0.01278682 | 0.00897174 |
| Omura's whale | *Balaenoptera omurai* | 0.23104473 | 0.2296745 |
| Pantropical spotted dolphin | *Stenella attenuata* | 0.18658059 | 0.16509789 |
| Pygmy sperm whale | *Kogia breviceps* | 0.35467093 | 0.24661996 |
| Risso's dolphin | *Grampus griseus* | 0.12651749 | 0.13388637 |
| Rough-toothed dolphin | *Steno bredanensis* | 0.10156076 | 0.15922095 |
| Short-finned pilot whale | *Globicephala macrorhynchus* | 0.10278151 | 0.04096735 |
| Sperm whale | *Physeter macrocephalus* | 0.112129 | 0.18694743 |
| Spinner dolphin | *Stenella longirostris* | 0.09876644 | 0.12081179 |
| Striped dolphin | *Stenella coeruleoalba* | 0.07255582 | 0.16655724 |
| Dugong | *Dugong dugon* | 0.29731408 | 0.90995943 |
